# Leveraging Existing Data from CMS-Linked Cohort Studies for the Advancement and Translation of Frailty Research

**DOI:** 10.1093/geroni/igaa057.2810

**Published:** 2020-12-16

**Authors:** Qian-Li Xue, Kristine Ensrud, Shari Lin

## Abstract

As population aging is accelerating rapidly, there is growing concern on how to best provide patient-centered care for the most vulnerable. Establishing a predictable and affordable cost structure for healthcare services is key to improving quality, accessibility, and affordability. One such effort is the “frailty” adjustment model implemented by the Centers for Medicare & Medicaid Services (CMS) that adjusts payments to a Medicare managed care organization based on functional impairment of its beneficiaries. Earlier studies demonstrated added value of this frailty adjuster for prediction of Medicare expenditures independent of the diagnosis-based risk adjustment. However, we hypothesize that further improvement is possible by implementing more rigorous frailty assessment rather than relying on self-report of ADL difficulties as used for the frailty adjuster. This is supported by the consensus and clinical observations that neither multimorbidity nor disability alone is sufficient for frailty identification. This symposium consists of four talks that leverage data from three CMS-linked cohort studies to investigate the utility of assessment of the frailty phenotype for predicting healthcare utilization and costs. Talk 1 and 2 use data from the NHATS cohort to assess healthcare utilization by frailty status in the general population and the homebound subset. Talk 3 and 4 use data from the MrOS study and the SOF study to investigate the impact of frailty phenotype on healthcare costs. Taken together, their findings highlight the potential of incorporating phenotypic frailty assessment into CMS risk adjustment to improve the planning and management of care for frail older adults.

